# Combined radiofrequency and topical exosomes in atrophic vulvar lichen sclerosus: A case report

**DOI:** 10.1002/ijgo.71178

**Published:** 2026-06-23

**Authors:** Luisa Mendonca de Oliveira, Narciso DiLorenzo Machado Dias, Jacqueline Maia Ferraz, Patricia da Silva Oliveira

**Affiliations:** ^1^ Instituto Multidisciplinar em Saúde Universidade Federal da Bahia Vitória da Conquista Brazil

**Keywords:** exosomes, lichen sclerosus, radiofrequency, vulvar disorders

## Abstract

Vulvar lichen sclerosus (VLS) is a chronic inflammatory dermatological‐gynecological condition. Conventional first‐line treatment comprises topical corticosteroids; however, many patients remain refractory. Energy‐based devices, including radiofrequency (RF), have shown promising results in VLS, with symptomatic and functional improvement. Exosomes are vesicles containing bioactive molecules involved in tissue regeneration; their lyophilized form, stabilized for topical application, has been combined with energy‐based therapies with satisfactory results in other dermatological conditions. To our knowledge, this is the first report of ablative RF combined with topical lyophilized exosomes for VLS. We present the case of a 38‐year‐old woman with biopsy‐confirmed VLS refractory to standard treatment, who underwent ablative fractional RF combined with topical lyophilized exosomes derived from amniotic fluid stem cells. The patient showed improvement in pruritus, dyspareunia, and sexual function, and reported satisfaction with the combined therapy.

Vulvar lichen sclerosus (VLS) is a chronic Th1‐predominant condition, affecting prepubertal and postmenopausal women, often associated with other immune‐mediated disorders such as Hashimoto's thyroiditis.^1^ VLS typically presents as hypopigmented areas, pruritus, dysuria, dyspareunia, and fissures, potentially progressing to anatomical changes, chronic pain and increased malignancy risk. Due to its complex treatment, early clinical diagnosis confirmed by histopathology is essential.^1^

Radiofrequency (RF) promotes tissue repair through thermal energy and improving collagen synthesis,^2^, ^3^, ^4^ while exosomes, extrapolated from general regenerative models, may facilitate dermal matrix remodeling, stimulate fibroblast activity, and attenuate the Th1‐predominant lymphocytic infiltrate characteristic of VLS.^5^, ^6^ Combining ablative RF with topical exosomes has shown synergistic effects in dermatologic conditions.^5^ To our knowledge, this is the first case report describing ablative RF combined with topical lyophilized exosomes for VLS. Ethical approval and patient consent were obtained (CEP/IMS/UFBA – CAAE 84216824.2.0000.5556).

A 38‐year‐old woman with Hashimoto's thyroiditis presented with a three‐year history of refractory VLS symptoms. Her diagnosis was confirmed by biopsy (Figure S1). Previous therapies, including clobetasol, pelvic physiotherapy, estriol, amitriptyline, and topical testosterone, provided limited relief. Partial improvement followed four non‐ablative radiofrequency (RF) sessions, yet dryness and dyspareunia persisted. Subsequently, she underwent three sessions of ablative RF (matrix tip, 20 mJ) combined with topical lyophilized exosomes derived from amniotic fluid stem cells. During this protocol, she maintained topical clobetasol and testosterone, as well as unchanged doses of estriol, amitriptyline, and physiotherapy (Figure S2, Table S1).

Significant clinical improvement followed: reduced hypochromia, improved skin turgor and elasticity and resolution of fissures (Figure 1). Symptoms were monitored using a Vulvovaginal Symptoms Questionnaire translated and adapted^7^ (Table S2). The patient reported decreased pruritus and dyspareunia, improved libido, and reduced frustration.

**FIGURE 1:**
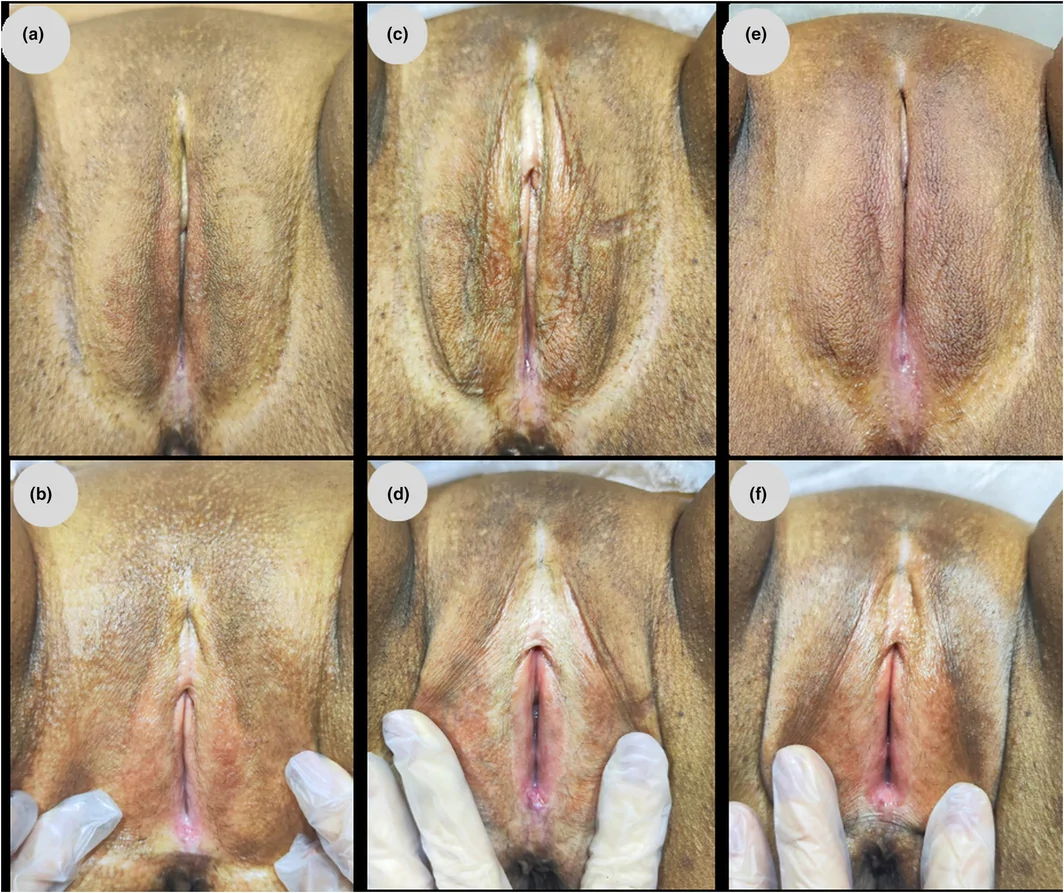
Progressive vulvar changes during combined therapy. (a–f) Before and immediately after each session.

Ablative RF induces neocollagenesis and neoelastogenesis,^2^, ^3^, ^4^ while exosomes, extrapolated from general regenerative models, may facilitate matrix remodeling and suppress proinflammatory cytokines.^5^, ^6^ These mechanisms in VLS remain hypothetical. Although clinical and anatomical improvements were observed, this single case cannot establish causality, and a placebo effect cannot be ruled out. Concurrent therapies and the limited follow‐up preclude definitive attribution of effect; nonetheless, the patient remains under active monitoring, with no recurrence documented at the last assessment in December 2025. Nevertheless, these preliminary findings suggest that this approach is feasible for refractory VLS, warranting further controlled trials to assess long‐term durability and recurrence risk.

## AUTHOR CONTRIBUTIONS

LMO: Conceptualization, investigation, analysis and draft of manuscript. NDLMD: Investigation, analysis, and draft of manuscript. JMF: Supervision, patient care, and data interpretation, PSO: Co‐supervision, histopathology, and manuscript. All authors approved and are accountable for the manuscript.

## FUNDING INFORMATION

The authors have nothing to report.

## CONFLICT OF INTEREST STATEMENT

The authors have no conflicts of interest to declare.

## Supporting information


**FIGURE S1:** Histopathology of fragment removed from the vulvar epidermis; patient diagnosed with vulvar atrophic lichen sclerosus. (a) Hypothrophy of the epidermis and absence of dermal papillae (white arrow), hyperemia, angiogenesis (dark green arrows), and vascular ectasia (blue arrows). (b) In the dermis, fibroblasts with aberrant nuclei (black arrows) and angiogenesis (dark green arrows). (c) Aberrant nuclei in keratinocytes (light green arrow) and presence of basal keratinocytes with vacuolar degeneration (yellow arrow). (d) Predominantly lymphocytic inflammatory infiltrate (white circles). Stain: H&E. Magnification: 400x.

**FIGURE S2:** Timeline of clinical progression and therapeutic interventions (2021–2024). The timeline illustrates the transition from conventional topical therapies and non‐ablative radiofrequency to the combined protocol of fractional ablative radiofrequency and topical exosomes. Abbreviations: RF, radiofrequency; VLS, vulvar lichen sclerosus.

**TABLE S1.** Detailed visit and follow‐up log.

**Table S2:** Patient responses and the administered questionnaire in Appendix A [Portuguese] and Appendix B [English]).

## Data Availability

Data sharing is not applicable to this article because no new data were created or analyzed in this study. The data that support the findings are available within the article and its supporting information (S1, S2, S3).
