# Supplementary material for: Combined radiofrequency and topical exosomes in atrophic vulvar lichen sclerosus: A case report
Source: Int J Gynaecol Obstet. 2026 Jun 23;175(3):1719–21. doi: 10.1002/ijgo.71178 (PMC13769337; doi:10.1002/ijgo.71178)
Supplement: Supplementary file 1 [file IJGO-175-1719-s002.docx]

**Table S1** - Detailed visit and follow‑up log.

| **Date** | **Visit / Note** | **Assessments / Findings** | **Interventions / Prescriptions** | **Patient-reported outcomes / Notes** |
| --- | --- | --- | --- | --- |
| 2021-07 | Initial presentation | Cutaneous atrophy of the vulva; clitoral and fourchette hypochromia; burial of ~2/3 of the labia minora | No specific prescription recorded at this visit | Patient reports long-standing symptoms (~3 years) |
| 2021-08 | Diagnostic confirmation | Biopsy: findings consistent with atrophic vulvar lichen sclerosus (epidermal atrophy, chronic lymphocytic infiltrate) | Clobetasol cream 0.5 mg/g – daily topical use prescribed | Diagnosis confirmed; counseling and treatment plan initiated |
| 2021-09 | Follow-up | Low-intensity nocturnal pruritus; otherwise, stable | Clobetasol 0.5 mg/g: alternate-day topical use + emollient (bepantol) | Symptom monitoring; partial control reported |
| 2021-10 | Clinical recurrence | Recurrence of pruritus, vaginal dryness and appearance of fissures | Clobetasol 0.5 mg/g: daily topical use in affected areas; alternate days in stable regions; (record also notes first non-ablative RF session at/near this period) | Worsening symptoms; fissures present |
| 2022-02 | Follow-up / Procedure | Worsening pruritus, change in skin texture, dyspareunia | 2nd non-ablative radiofrequency session performed | Symptoms persisted despite prior measures |
| 2022-03 | Follow-up | Improved lubrication and reduced dyspareunia reported | No change in systemic therapy recorded | Patient reports symptomatic improvement |
| 2022-04 | Acute event | Post-coital fissures observed | Topical collagenase 0.6 U/g prescribed | Local wound care advised |
| 2022-06 | Procedures and medications | Persistent post-coital fissures | 3rd and 4th non-ablative RF sessions performed; collagenase 0.6 U/g and chloramphenicol 0.01 g/g prescribed as needed | Partial healing; ongoing management |
| 2022-07 | Recovery / Adjuncts | Improvement in fissures; healed perineum observed | Initiated pelvic physiotherapy; estriol 0.1 mg/g vaginal cream; continue clobetasol 0.5 mg/g daily; amitriptyline 10 mg started | Noted clinical improvement in fissure healing |
| 2022-09 | Persistent symptoms | Partial improvement in pruritus and hypochromia; slight; deep dyspareunia, sinusorrhagia, vaginal dryness and urinary incontinence | Testosterone propionate 5% topical application to clitoral area on alternate days added | Symptoms partially responsive; sexual function affected |
| 2022-10 | Follow-up | Improvement in pruritus; ongoing sinusorrhagia and genital fissures | Continuing topical regimens and pelvic physiotherapy as indicated | Monitoring; symptomatic changes documented |
| 2024-03 | Maintenance visit | Stable condition noted | Continue testosterone propionate 5% topical application to clitoral area on alternate days | Stable clinical status reported by patient |
| 2024-09 | Initiation of combined protocol | Stable condition prior to intervention | Start of radiofrequency combined with lyophilized exosomes (protocol initiated) | Consent obtained; baseline questionnaires recorded |
| 2024-11 | Post-treatment assessment | Improved libido; reduced intensity of dyspareunia and sinusorrhagia | Radiofrequency + lyophilized exosomes sessions ongoing/complete as per protocol | Clinical improvement noted; check questionnaire scores in Table S2B |
| 2024-12 | Final recorded follow-up (last available) | Reduced pruritus, tissue friability/fissures, dryness and discharge | Radiofrequency combined with lyophilized exosomes – ongoing maintenance/assessments | Marked symptomatic and anatomical improvement documented |

Note: Dates are presented in YYYY-MM format to preserve clinical chronology. All personal identifiers have been removed to ensure patient confidentiality. Outcomes in 2024 refer to the post-intervention assessment period for the combined protocol. Abbreviations: RF, radiofrequency; VLS, vulvar lichen sclerosus; YYYY-MM, year-month.
