# Supplementary material for: Combined radiofrequency and topical exosomes in atrophic vulvar lichen sclerosus: A case report
Source: Int J Gynaecol Obstet. 2026 Jun 23;175(3):1719–21. doi: 10.1002/ijgo.71178 (PMC13769337; doi:10.1002/ijgo.71178)
Supplement: Supplementary file 2 [file IJGO-175-1719-s003.docx]

**Table S2 - Patient responses to the questionnaire**

|  | **Symptom Intensity** | | |
| --- | --- | --- | --- |
| **Questions regarding symptoms** | **S1** | **S2** | **S3** |
| 1. During the last week, did you experience vulvar itching? | Yes, 7 | Yes, 4 | Yes, 6 |
| 2. During the last week, did you experience burning or stinging in the vulva? | No | No | No |
| 3. During the last week, did you experience vulvar pain? | No | No | No |
| 4. During the last week, did you experience irritation or notice redness in the vulva? | No | No | Yes, 5 |
| 5. During the last week, did you experience vulvar dryness? | Yes, 5 | No | No |
| 6. During the last week, did you feel that your vulvar skin was fragile (were there any fissures/bleeding)? | Yes, 8 | Yes, 3 | No |
| 7. During the last week, did you notice any vaginal discharge? | Yes, 5 | No | No |
| 8. During the last week, did you notice any vaginal odor? | No | No | No |
| **Emotions** | | | |
| 9. Do you worry about your vulvar symptoms? (For example, that they will spread, worsen, scar, etc.) | Yes, 10 | Yes, 9 | Yes, 10 |
| 10. Do you feel that the appearance of your vulva has a negative impact on your self-perception or self-image? | Yes, 10 | No | No |
| 11. Do you feel frustrated with your vulvar symptoms? | Yes, 8 | Yes, 3 | Yes, 6 |
| 12. Do you feel embarrassed about your vulvar symptoms? | No | No | No |
| 13. Do you feel that your vulvar symptoms affect your interactions with other people? | No | No | No |
| 14. Do you feel that your vulvar symptoms affect your desire to be with other people? | No | No | No |
| **Impact on Life** | | | |
| 15. Do your vulvar symptoms make it difficult for you to show affection? | No | No | No |
| 16. Do your vulvar symptoms affect your daily activities? | No | No | No |
| 17. Do your vulvar symptoms affect your desire for intimate contact with another person? | Yes, 7 | No | No |
| 18. Are you sexually active with an intimate partner? | Yes | Yes | Yes |
| **Social Impact** | | | |
| 19. Do your vulvar symptoms negatively affect your intimate contact with another person? | Yes, 9 | No | No |
| 20. Do your vulvar symptoms cause pain during sexual activity? | Yes, 9 | Yes, 3 | Yes, 6 |
| 21. Do your vulvar symptoms cause dryness during sexual activity? | Yes, 6 | Yes, 5 | No |
| 22. Do your vulvar symptoms cause bleeding after sexual activity? | Yes, 5 | No | Yes, 3 |

Notes: Symptoms were rated by the patient on a scale of 0 to 10 (where 0 is absent and 10 is maximum intensity) 15 days after each therapeutic session. "No" indicates the absence of the symptom during the assessed week. Abbreviations: S1, Session 1; S2, Session 2; S3, Session 3.

**Appendix A : Administered Questionnaire (in Portuguese)**

**Identificador do paciente:** ___________________

**Data (YYYY‑MM‑DD):** ___________________

**Sessão / Avaliação:** ☐ Pré‑tratamento ☐ Após 1ª sessão ☐ Após 2ª sessão ☐ Após 3ª sessão

**Instruções:** Para cada item, assinale "Sim" ou "Não". Se assinalar "Sim", indique a intensidade do sintoma de 1 a 10 (1 = intensidade mínima, 10 = intensidade máxima) considerando a última semana e respondendo 15 dias após a sessão quando aplicável.

#### **Sintomas**

1. Na última semana, você sentiu prurido vulvar? ☐ Não ☐ Sim — intensidade: ____ /10

2. Na última semana, você sentiu queimação ou ardor na vulva? ☐ Não ☐ Sim — intensidade: ____ /10

3. Na última semana, você sentiu dor vulvar? ☐ Não ☐ Sim — intensidade: ____ /10

4. Na última semana, notou irritação ou vermelhidão na vulva? ☐ Não ☐ Sim — intensidade: ____ /10

5. Na última semana, sentiu ressecamento vulvar? ☐ Não ☐ Sim — intensidade: ____ /10

6. Na última semana, sentiu que sua pele vulvar estava frágil (fissuras/sangramento)? ☐ Não ☐ Sim — intensidade: ____ /10

7. Na última semana, notou corrimento vaginal? ☐ Não ☐ Sim — intensidade: ____ /10

8. Na última semana, notou odor vaginal? ☐ Não ☐ Sim — intensidade: ____ /10

#### **Emoções / Percepção**

9. Você se preocupa com seus sintomas vulvares (por exemplo, que eles piorem ou deixem cicatriz)? ☐ Não ☐ Sim — intensidade: ____ /10

10. A aparência da sua vulva tem impacto negativo na sua autoimagem? ☐ Não ☐ Sim — intensidade: ____ /10

11. Você se sente frustrada com seus sintomas vulvares? ☐ Não ☐ Sim — intensidade: ____ /10

12. Você se sente envergonhada pelos seus sintomas vulvares? ☐ Não ☐ Sim — intensidade: ____ /10

13. Você sente que os sintomas afetam suas interações com outras pessoas? ☐ Não ☐ Sim — intensidade: ____ /10

14. Os sintomas afetam seu desejo de estar com outras pessoas? ☐ Não ☐ Sim — intensidade: ____ /10

#### **Impacto na vida diária e sexualidade**

15. Seus sintomas dificultam demonstrar afeto (ex.: abraços, carinho)? ☐ Não ☐ Sim — intensidade: ____ /10

16. Seus sintomas afetam suas atividades diárias? ☐ Não ☐ Sim — intensidade: ____ /10

17. Seus sintomas afetam seu desejo por contato íntimo? ☐ Não ☐ Sim — intensidade: ____ /10

18. Você está sexualmente ativa com parceiro(a)? ☐ Não ☐ Sim

19. Seus sintomas afetam negativamente o contato íntimo com outra pessoa? ☐ Não ☐ Sim — intensidade: ____ /10

20. Seus sintomas causam dor durante a atividade sexual? ☐ Não ☐ Sim — intensidade: ____ /10

21. Seus sintomas causam secura durante a atividade sexual? ☐ Não ☐ Sim — intensidade: ____ /10

22. Seus sintomas causam sangramento após a atividade sexual? ☐ Não ☐ Sim — intensidade: ____ /10

**Appendix B: Administered Questionnaire (in English – for review)**

**Patient ID:** ___________________

**Date (YYYY‑MM‑DD):** ___________________

**Session / Assessment:** ☐ Baseline ☐ After Session 1 ☐ After Session 2 ☐ After Session 3

**Instructions:** For each item, mark "No" or "Yes". If "Yes", indicate the symptom intensity from 1 to 10 (1 = minimal intensity, 10 = maximal intensity) referring to the last week and, when applicable, assessed 15 days after each session.

#### **Symptoms**

1. During the last week, did you experience vulvar itching? ☐ No ☐ Yes — intensity: ____ /10

2. During the last week, did you experience burning or stinging in the vulva? ☐ No ☐ Yes — intensity: ____ /10

3. During the last week, did you experience vulvar pain? ☐ No ☐ Yes — intensity: ____ /10

4. During the last week, did you notice irritation or redness in the vulva? ☐ No ☐ Yes — intensity: ____ /10

5. During the last week, did you experience vulvar dryness? ☐ No ☐ Yes — intensity: ____ /10

6. During the last week, did you feel that your vulvar skin was fragile (fissures/bleeding)? ☐ No ☐ Yes — intensity: ____ /10

7. During the last week, did you notice any vaginal discharge? ☐ No ☐ Yes — intensity: ____ /10

8. During the last week, did you notice any vaginal odor? ☐ No ☐ Yes — intensity: ____ /10

#### **Emotions / Self‑perception**

9. Do you worry about your vulvar symptoms (e.g., that they will worsen or scar)? ☐ No ☐ Yes — intensity: ____ /10

10. Do you feel that the appearance of your vulva has a negative impact on your self‑image? ☐ No ☐ Yes — intensity: ____ /10

11. Do you feel frustrated with your vulvar symptoms? ☐ No ☐ Yes — intensity: ____ /10

12. Do you feel embarrassed about your vulvar symptoms? ☐ No ☐ Yes — intensity: ____ /10

13. Do your symptoms affect your interactions with other people? ☐ No ☐ Yes — intensity: ____ /10

14. Do your symptoms affect your desire to be with other people? ☐ No ☐ Yes — intensity: ____ /10

#### **Impact on daily life and sexuality**

15. Do your symptoms make it difficult for you to show affection? ☐ No ☐ Yes — intensity: ____ /10

16. Do your symptoms affect your daily activities? ☐ No ☐ Yes — intensity: ____ /10

17. Do your symptoms affect your desire for intimate contact with another person? ☐ No ☐ Yes — intensity: ____ /10

18. Are you sexually active with an intimate partner? ☐ No ☐ Yes

19. Do your symptoms negatively affect your intimate contact with another person? ☐ No ☐ Yes — intensity: ____ /10

20. Do your symptoms cause pain during sexual activity? ☐ No ☐ Yes — intensity: ____ /10

21. Do your symptoms cause dryness during sexual activity? ☐ No ☐ Yes — intensity: ____ /10

22. Do your symptoms cause bleeding after sexual activity? ☐ No ☐ Yes — intensity: ____ /10

**Note:** Note: Questionnaire adapted from Alem et al. (2022). This adaptation did not undergo formal psychometric validation. Interpret results with caution.

Alem VC, de Oliveira AC, De‑Luca MP, de Oliveira MS, Eleutério Junior J, Amaral RL. Tradução e adaptação transcultural do Vulvovaginal Symptoms Questionnaire para a língua portuguesa. Rev Bras Ginecol Obstet. 2022;44(6):610–618. doi:10.1055/s-0042-1744288De
