# Supplementary material for: Combined radiofrequency and topical exosomes in atrophic vulvar lichen sclerosus: A case report
Source: Int J Gynaecol Obstet. 2026 Jun 23;175(3):1719–21. doi: 10.1002/ijgo.71178 (PMC13769337; doi:10.1002/ijgo.71178)
Supplement: Supplementary file 3 [file IJGO-175-1719-s004.docx]

**Figure S2** - Timeline of clinical progression and therapeutic interventions (2021–2024). The timeline illustrates the transition from conventional topical therapies and non-ablative radiofrequency to the combined protocol of fractional ablative radiofrequency and topical exosomes. Abbreviations: RF, radiofrequency; VLS, vulvar lichen sclerosus.
